# Cognitive function in pituitary adenoma patients: A cross-sectional study

**DOI:** 10.1371/journal.pone.0309586

**Published:** 2024-09-06

**Authors:** David Krabbe, Katharina S. Sunnerhagen, Daniel S. Olsson, Tobias Hallén, Oskar Ragnarsson, Thomas Skoglund, Gudmundur Johannsson

**Affiliations:** 1 Department of Rehabilitation Medicine, Sahlgrenska University Hospital, Gothenburg, Sweden; 2 Department of Clinical Neuroscience, Institute of Neuroscience and Physiology, Sahlgrenska Academy, University of Gothenburg, Gothenburg, Sweden; 3 Department of Medicine, Sahlgrenska University Hospital, Gothenburg, Sweden; 4 Department of Internal Medicine and Clinical Nutrition, Institute of Medicine, Sahlgrenska Academy, University of Gothenburg, Gothenburg, Sweden; 5 Late-Stage Clinical Development, Cardiovascular, Renal and Metabolism (CVRM), BioPharmaceuticals R&D, AstraZeneca, Mölndal, Sweden; 6 Department of Neurosurgery, Sahlgrenska University Hospital, Gothenburg, Sweden; 7 Wallenberg Centre for Molecular and Translational Medicine, Institute of Medicine, Sahlgrenska Academy, University of Gothenburg, Gothenburg, Sweden; Hamad Medical Corporation, QATAR

## Abstract

Various factors may affect cognition in patients with pituitary adenoma, including size and extension of the tumor, degree of pituitary hormone deficiencies, and treatment of the tumor, most often being transsphenoidal surgery (TSS). The aim of this cross-sectional study was to evaluate cognitive function in patients with clinically significant pituitary adenoma and to identify factors influencing cognition. Sixty-eight patients with pituitary adenoma were included. Of these, 31 patients were evaluated before TSS and 37 patients 12 months following TSS. Cognitive function was evaluated by using the Repeatable Battery for the Assessment of Neuropsychological Status. Patients had lower mean scores on cognitive assessment compared to age-adjusted normative data. Variability in cognition, analyzed by linear regression analysis, was explained by sex, educational level, and self-perceived fatigue, but not by pituitary hormone deficiencies, diabetes insipidus, or surgical treatment. Our results are in line with previous findings, namely that pituitary adenoma affects cognition. To better evaluate the factors affecting cognition, longitudinal studies are recommended. Such studies would allow for within-individual comparisons, effectively controlling for the considerable influence of sex and education on test results.

## Introduction

Pituitary adenomas are benign tumors that may cause hypopituitarism, various syndromes related to hormone hypersecretion, and visual impairment [1]. Non-functioning pituitary macroadenomas with suprasellar extension may affect cognition due to their impact on nearby neuronal structures such as the mammillary bodies [2]. Further, macroadenomas with chiasm compression may affect the suprachiasmatic nuclei and regulation of sleep-wake cycles [3]. Except for prolactinomas, transsphenoidal surgery (TSS) is the primary treatment of choice for most symptomatic pituitary adenomas and is considered as being associated with a low risk of complications [4]. Knowledge about whether TSS *per se* impacts cognition is limited. A systematic review published in 2017 reported that executive functions and memory may be affected after TSS, with impairments in verbal memory being most common [5]. Other studies have not shown any adverse effect of surgery on cognition [6] as well as cognitive improvement during postoperative follow-up [7], which might be attributed to improvement in endocrine function and/or adequate endocrine replacement therapy. The reasons for cognitive dysfunction in patients with pituitary adenomas are likely to be multifactorial, including choice of primary treatment, size and extension of the tumor, and degree of pituitary hormone deficiency. Although it has been addressed previously, cognitive function and the factors affecting it still have important implications for many patients with pituitary adenoma and need further clarification.

In this explorative cross-sectional study, we aimed at evaluating cognition in patients with pituitary adenomas and to identify factors that are related with it.

## Material and methods

### Design

This was a cross-sectional study with consecutive recruitment of adult patients participating in the Gothenburg Pituitary Tumor Study (GoPT-study; project number 161671 in research and development in Sweden), a large prospective study that enrolled patients scheduled for pituitary surgery at Sahlgrenska University Hospital, the sole provider of neurosurgical services for just over 1.9 million people in the western region of Sweden [8]. Patients were scheduled for first TSS for pituitary adenoma or TSS reoperation for pituitary adenoma regrowth. During the first year after inclusion in the GoPT-study, cognition was assessed on one occasion: either before surgery or at 12 months follow-up. Thus, for one subgroup of patients recruited to the present study, TSS was not part of the recent medical history and for the other it was (1 year prior). The cross-sectional design was chosen to explore different factors associated with cognition, one of them being point in time for assessment. From the findings of this explorative study, additional studies on cognition within the GoPT-study could be designed.

### Participants

**Fig 1:**
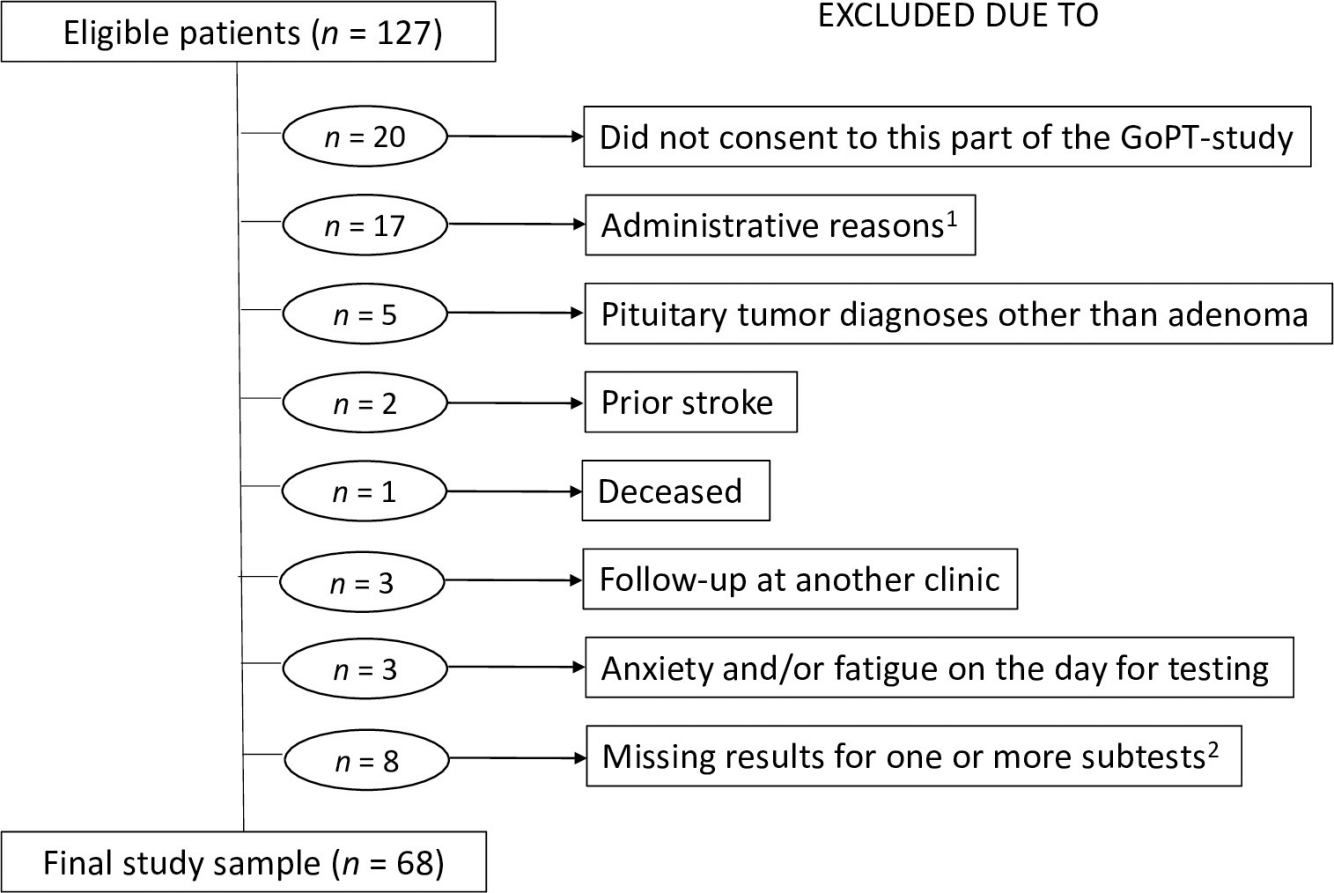
Attrition diagram. ^1^The scheduled visit could not be coordinated for cognitive testing. ^2^Missing results due to patients having impaired sight (*n* = 2), difficulties in writing due to a venous catheterization (*n* = 2), unaccustomed to the Swedish language (*n* = 3), and lack of one of the test forms at the time for testing (*n* = 1).

Sixty-eight patients were consecutively recruited from 30 September 2016 to 5 September 2018 from a group of 127 patients participating in the prospective GoPT-study (Fig 1). Four patients scheduled for pituitary adenoma surgery were not included in the GoPT-study due to acute surgery (*n* = 2), deceased before surgery (*n* = 1), and administrative reason (*n* = 1). Reasons for exclusion from the present study are recorded in Fig 1. The patients included did not differ significantly from those who were excluded with respect to sex (*p* = 0.22) or age (*p* = 0.56).

For 10 patients in the subgroup with cognitive assessment before TSS the reason for being scheduled for surgery was tumor regrowth after previous surgery (Table 1) with a median time interval between previous and current surgery and cognitive assessment of 8 years (range 3–17 years). Further, 11 patients in the subgroup with cognitive assessment before TSS had anterior pituitary insufficiencies that were not substituted. These included four patients with known growth hormone deficiency, six with sex steroid deficiency, and two with mild central hypothyroidism. In total, 10 patients had chronic diabetes insipidus requiring treatment with desmopressin. Three patients had undergone radiotherapy before the cognitive assessment: two patients (7 and 18 years prior, respectively) in the subgroup tested before TSS and one patient (1 year prior) in the subgroup tested after TSS.

**Table 1:** Demographic and disease characteristics for the total population as well as for subgroups based on time of evaluation.

Characteristic	Total (*n* = 68)	Preop (*n* = 31)	Postop (*n* = 37)	*P*-value
**Sex, *n* (%)**				0.49
** Women**	32 (47)	16 (52)	16 (43)	
** Men**	36 (53)	15 (48)	21 (57)	
**Mean age, yr (SD)**	57 (15)	59 (15)	55 (16)	0.26
**Mean education duration, yr (SD)**	12.7 (3.2)	12.5 (3.3)	12.9 (3.1)	0.65
**Type of surgery scheduled, *n* (%)**				0.21
** Primary surgery**	51 (75)	21 (68)	30 (81)	
** Reoperation** ^**a**^	17 (25)	10 (32)	7 (19)	
**Pituitary adenoma type, *n* (%)**				0.89
** Non-functioning**	51 (75)	23 (74)	28 (76)	
** Functioning**	17 (25)	8 (26)	9 (24)	
** **Acromegaly	8 (12)	5 (16)	3 (8)	
** **Cushing’s disease	5 (7)	2 (6)	3 (8)	
** **Prolactinoma	3 (4)	1 (3)	2 (5)	
** **Thyrotropinoma	1 (1)	0	1 (3)	
**Pituitary insufficiency, *n* (%)**				0.37
** None**	19 (28)	7 (23)	12 (32)	
** Any**	49 (72)	24 (77)	25 (68)	
** **Thyroid hormone	29 (43)	15 (48)	14 (38)	
** **Sex steroids	28 (41)	12 (39)	16 (43)	
** **Growth hormone	27 (40)^b^	10 (32)^b^	17 (46)	
** **Cortisol	21 (31)	8 (26)	13 (35)	
**Diabetes insipidus, *n* (%)**	10 (15)	6 (19)	4 (11)	

^a^Due to tumor regrowth.

^b^Presence of growth hormone deficiency not known for all patients before surgery.

*Note*. Pearson’s chi-square test was used for comparison of sex, surgical type (reoperation), pituitary adenoma type (functioning), and pituitary insufficiency (any), Mann-Whitney *U*-test for age, and *t*-test for education duration between subgroups.

*Abbreviations*: postop, 1-yr postoperatively; preop, preoperatively; SD, standard deviation; yr, year.

### Ethics

The study was approved by the regional ethical review board in Gothenburg, Sweden (Dnr: 387–15, T682-16). All participants gave written informed consent before participating in any part of the study.

### Variables and measures

Patient demographics including sex, age, and educational level (years of education) were recorded. Tumor type was determined based on clinical presentation, biochemical evaluation, and histopathological examination. Pituitary surgery was recorded as primary or reoperation due to tumor regrowth. All surgeries were endoscopic TSS and always performed by an ear, nose, and throat surgeon together with a neurosurgeon. Anterior pituitary hormone deficiencies were classified in the case of one or more of the following hormones: thyroid hormone, growth hormone, sex steroids, or cortisol [9]. Diabetes insipidus was defined as polyuria requiring chronic desmopressin replacement. Endocrine evaluation for pituitary insufficiency and diabetes insipidus was performed before surgery, within 1 week after surgery, and at 3–6 months postoperatively. Evaluation of growth hormone secretion was performed at 6–12 months postoperatively.

The Repeatable Battery for the Assessment of Neuropsychological Status (RBANS) was used for evaluation of cognitive function. RBANS, originally developed for the assessment of dementia, has been used in various medical settings [10], including assessment of cognitive deficits in adult patients with primary brain tumors [11]. In our study, the Swedish version of RBANS was used. The normative data for the Swedish version of RBANS includes 454 individuals aged 20–89 years from the normal population in Sweden, Denmark, and Norway. The selection of the sample aimed at corresponding with the age distribution and educational level of the normal population. To ensure representativeness, the selection was based on official statistics from these countries. Exclusion criteria at recruitment were, for example, medical or psychiatric conditions that might affect cognitive function and lack of sufficient language skills [12]. RBANS consists of 12 subtests. Each subtest contributes to one of five cognitive domains or indices: immediate memory, visuospatial/constructional, language, attention, and delayed memory. The index scores are corrected for age and can be converted into a total index score, indicating the general cognitive functioning of the examinee. For domain and total indices, the mean is 100 and standard deviation (SD) is 15. Actual testing time is 20–30 minutes. Two parallel versions of RBANS are available (A and B).

Fatigue was assessed using the Swedish Multidimensional Fatigue Inventory (MFI-20) [13]. In this questionnaire, the examinees rate to what extent statements regarding different dimensions of fatigue relate to them. MFI-20 has five subscales, each including four statements. The subscales are general fatigue, physical fatigue, mental fatigue, activity, and motivation. Answers are given on a five-point scale. Each subscale has a possible score range of 4–20, with higher values indicating higher levels of fatigue. Results from the general fatigue subscale were used in this study.

### Setting

Test sessions took place in a quiet office, either at the Department of Neurosurgery the day before surgery or at the Department of Endocrinology 12 months after surgery. All patients were tested with RBANS by the same neurorehabilitation psychologist (DK). MFI-20 was administered by a research nurse, with the patients completing their questionnaires on a digital tablet or on paper.

### Statistics

Data were analyzed using SPSS version 27 (IBM Corp., Armonk, NY). Mann-Whitney *U*-test was used for non-normally distributed data, *t*-test for normally distributed data, and Pearson’s chi-square test for categorical data. Independent variables with *p*-values < 0.25 in univariable linear regression analyses were selected for a multivariable linear regression analysis. Subgroup analyses were performed based on whether evaluation was before or after surgery.

## Results

### Study sample

A total of 68 patients were included in the final analysis (Table 1). For women and men respectively, mean age at test administration was 56 and 58 years (*p* = 0.66), mean education level was 12.9 and 12.6 years (*p* = 0.64), and mean general fatigue was 14.1 (5.5) and 11.0 (5.5; *p* = 0.024).

### Cognitive function in pituitary adenoma patients

RBANS total and domain index means for the total sample and subgroups of patients are presented in Table 2. Mean RBANS total index score for patients with intact pituitary function was not different from normative data, whereas patients with any pituitary hormone deficiency had lower scores compared to normative data.

**Table 2:** RBANS total and domain index mean [95% confidence interval] for the total sample and for subgroups.

	Total index	Immediate memory	Visuospatial/ constructional	Language	Attention	Delayed memory
**Total sample (n = 68)**	87 [83–91]	90 [85–94]	91 [88–93]	96 [92–99]	95 [91–99]	87 [83–91]
** Women (n = 32)**	94 [88–100]	98 [92–104]	89 [86–93]	100 [95–106]	99 [93–105]	92 [87–98]
** Men (n = 36)**	82 [76–87]	82 [76–87]	92 [88–96]	91 [86–96]	91 [87–96]	82 [76–88]
**Intact pituitary function (n = 19)**	92 [81–102]	91 [79–103]	94 [88–99]	97 [89–104]	101 [93–110]	89 [81–97]
**Pituitary hormone deficiency (n = 49)**	86 [81–90]	89 [85–93]	90 [87–92]	95 [91–99]	93 [89–96]	86 [81–91]
** Thyroid hormone deficiency (n = 29)**	85 [79–90]	89 [83–95]	87 [84–91]	96 [91–101]	93 [88–97]	84 [78–91]
** Sex steroid deficiency (n = 28)**	83 [77–89]	84 [78–89]	91 [87–95]	94 [88–100]	94 [88–99]	80 [74–87]
** Growth hormone deficiency (n = 27)**	89 [83–94]	95 [89–101]	91 [86–95]	95 [90–101]	94 [89–100]	88 [82–94]
** Adrenal insufficiency (n = 21)**	84 [77–90]	88 [82–95]	87 [82–92]	94 [87–100]	91 [85–97]	85 [77–94]
**Diabetes insipidus (n = 10)**	82 [69–96]	86 [71–101]	85 [77–92]	89 [79–98]	99 [86–111]	83 [74–92]

*Abbreviations*: RBANS, Repeatable Battery for the Assessment of Neuropsychological Status.

RBANS total index mean was not different between patients with functioning pituitary adenoma (FPA) and non-functioning pituitary adenoma (NFPA; *p* = 0.63).

The impact of different factors on RBANS total index score was investigated by linear regression analyses (Table 3). The *p*-values for diabetes insipidus and reoperation were above 0.25 in univariable analyses and thus these factors were not included in multivariable analysis. The impact of pituitary insufficiency and recent TSS were not statistically significant in the multivariable analysis in contrast to the impact of sex, education, and fatigue. Female sex and education level were positively associated with RBANS total index score and general fatigue was negatively associated with the RBANS total index score.

**Table 3:** Regression analysis of factors associated with RBANS total index score.

Covariate	Univariable analysis (*n* = 68)		Multivariable analysis (*n* = 63)	
	Coefficient (95% CI)	*P*-value	Coefficient [95% CI]	*P*-value
**Sex (women are reference)**	–12.14 [–19.65, –4.64]	0.002	–14.87 [–22.09, –7.65]	< 0.001
**TSS (0 = no, 1 = yes)**	5.98 [–1.95, 13.92]	0.14	5.89 [–1.23, 13.00]	0.11
**General fatigue** ^**a**^	–0.64 [–1.36, 0.09]	0.087	–1.08 [–1.75, –0.40]	0.002
**Education level**	1.22 [–0.02, 2.46]	0.054	1.38 [0.27, 2.49]	0.015
**Diabetes insipidus (0 = yes, 1 = no)**	5.81 [–5.44, 17.05]	0.31	ND	ND
**Pituitary insufficiency (0 = yes, 1 = no)**	5.94 [–2.89, 14.77]	0.19	1.16 [–6.52, 8.84]	0.77
**Reoperation (0 = yes, 1 = no)**	–5.26 [–14.44, 3.93]	0.26	ND	ND

^a^*n* = 63. Five patients had missing data.

*Abbreviations*: CI, confidence interval; ND, not done (as *p* >0.25 on univariable analysis); RBANS, Repeatable Battery for the Assessment of Neuropsychological Status; TSS, transsphenoidal surgery.

### Analyses of subgroups based on whether evaluation was performed before or after TSS

**Fig 2:**
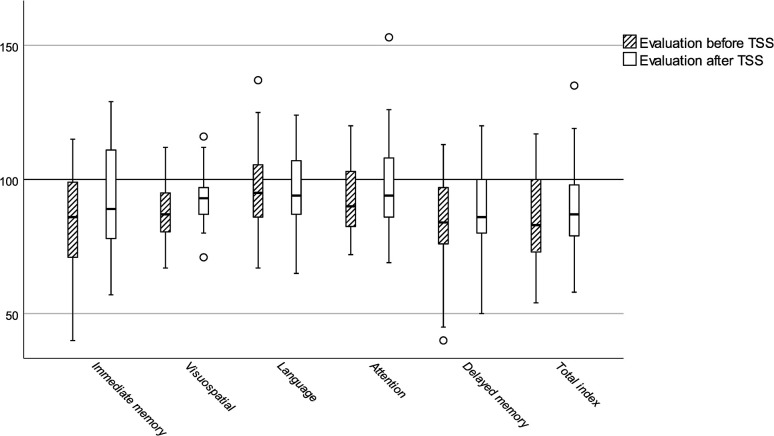
Box and whisker plot of repeatable battery for the assessment of neuropsychological status domain and total index scores. For normative data, mean is 100 and standard deviation is 15 for domain and total index scores.

Sex, age, educational level, tumor etiology, and number of reoperations did not differ between patients tested before TSS and patients tested after TSS (Table 1). Mean (SD) general fatigue derived from MFI-20 was 13.7 (5.0) for the subgroup tested before and 11.5 (6.1) for the subgroup tested after TSS (*p* = 0.15). RBANS subdomain and total index scores by subgroup are reported in Fig 2. There was no significant difference between subgroups in terms of RBANS total index score (*p* = 0.15). Lower mean RBANS total index scores were seen for both the subgroup tested before (84, 95% CI 78–90) and the subgroup tested after TSS (90, 95% CI 84–96) compared to normative data [12].

## Discussion

In this cross-sectional study, cognitive assessments using RBANS revealed that patients with pituitary adenoma scored lower compared to age-matched normative data. The most significant differences were observed in the memory domains, where patients exhibited the lowest mean scores. Five of six subtests that contribute to the memory indices in RBANS measure different aspects of verbal memory and the indices in turn contribute to the total index. Thus, to a considerable extent, the total score index is based on cognitive functions that may be especially sensitive to pituitary adenoma and its treatment.

Variability in the RBANS total score was not influenced by whether cognitive assessment was performed before or after TSS, but instead mainly by sex, educational level, and self-perceived fatigue. Furthermore, subgroup analysis showed no difference in RBANS total index score between the subgroup of patients tested before and the subgroup tested after TSS.

Transient and permanent diabetes insipidus are complications of TSS for pituitary adenoma. The risk of permanent diabetes insipidus has been shown to increase with increased tumor diameter [14] and craniocaudal diameter [15]. We hypothesized that tumor characteristics associated with a higher risk of permanent diabetes insipidus following surgery might also be associated with a higher risk for cognitive impairment. However, chronic diabetes insipidus did not significantly affect the variability in cognitive function. A weakness in this analysis of the current study is the fact that it was mostly women who had diabetes insipidus and women overall had higher RBANS total index scores.

Women also reported higher fatigue scores than men. This is probably related to factors other than pituitary adenoma or its treatment as higher scores on the general fatigue subscale of MFI-20 have been found among women than in men of the Swedish general population [16]. Further, women have been shown to score higher than men on most subtests of normative RBANS data for older adults, although most of these differences were of a small effect size [17]. Although such differences may be a part of the sex difference seen in the present study, other reasons are unknown but might be related to a different impact of pituitary hormone deficiency in women. When RBANS domain scores were divided by type of pituitary hormone deficiency, the lowest means were observed for the memory domains in the male subgroup with sex steroid deficiency. A previous study [18] noted more pronounced memory impairments in male patients with pituitary adenoma, suggesting that hormone dysfunction could be the primary cause. The impact of education level was expected as education level has previously been shown to influence all RBANS domain index scores [19].

It has been shown that cortisol [20, 21], growth hormones [22, 23], and thyroid hormones [24] may affect brain functions such as cognition. Comparative analysis with normative data, considering the presence of pituitary hormone deficiency, showed that RBANS total index scores were higher when pituitary deficiency was absent. Despite this, no contribution of pituitary insufficiency was seen in the regression analyses. Our study did not, however, have the power and was not designed to analyze the impact of each individual hormone deficiency and its replacement on cognition. For instance, a majority of the patients in the subgroup with pituitary hormone deficiency had more than one deficiency, which limits the possibilities to make direct comparisons between different hormone deficiencies. Moreover, in the subgroup that was tested before undergoing TSS, not all endocrine deficiencies had been adequately treated during the study period, a factor which could influence their cognitive function and levels of fatigue. No significant difference in RBANS total index mean was observed between patients with FPA and NFPA. The study’s limited sample size and the diversity of pituitary adenoma types included prevented an analysis of how different hormone hypersecretions impact the results. A larger group of patients with somatotroph adenomas was included in a prospective longitudinal study [25], and no significant difference in cognitive function was observed between this group and patients with NFPA. However, improvements were seen for patients with NFPA at retesting at 3 months after surgery, whereas no significant changes were observed for patients with somatotroph adenoma, suggesting that overproduction of growth hormone might be associated with specific cognitive deficits [25].

One of the aims of the study was to identify factors related to cognition in patients with pituitary adenoma. Concerning TSS, a confounder to consider in our study is that the situation for the patients is very different for the group tested before surgery compared to those tested 12 months after surgery. It could be argued that testing before surgery had a negative impact on their performance due to stress or other distractions. An assessment of the eligibility for participation in relation to anxiety and other mental states was always conducted by the psychologist administrating RBANS. Patients often reported that they found the cognitive testing interesting and that they appreciated the distraction it brought. In a previous study [26] of patients with various brain tumors referred for neuropsychological assessment before surgery (2–3 days), the findings did not confirm that distress has a strong negative impact on objective cognitive functioning. Another study [27] found that patients with very different emotional states had different test results. However, it was proposed that for the majority of neuro-oncological patients, evaluations using common neuropsychological measures are valid when conducted in the preoperative phase (1–8 days prior to surgery).

Another limitation is that instead of a healthy control group being specifically recruited and matched to the patients, test performance was related to previously developed normative data [12]. Differences in cognitive performance between men and women in the study sample, minor differences between the study and normative data in sex distribution, and also, possibly, in test protocol scoring may influence relations between results for patients and normative data.

The dispersion of the results within the subgroups of patients seems considerable in relation to any difference between the means of the subgroup tested before and the subgroup tested after TSS. As shown previously [28], individual courses after surgery can be markedly different, although the mean group level showed no change over time. In our study, the individual courses could not be followed. Concerning evaluation of effects of TSS on cognition, the cross-sectional design of this study is a major limitation. To be able to infer causality, and given the strong impact of sex and education level on cognition, a preferable study design would be longitudinal, comparing patients with themselves.

## Conclusion

In this study, fatigue, sex, and education levels were more important compared to TSS regarding variability in RBANS total index score. Since sex and education level had a strong impact, we propose that longitudinal approaches using within-individual comparison are used for evaluation of factors affecting cognition among patients with pituitary adenoma.
